# De novo pathogenic variant in *SETX* causes a rapidly progressive neurodegenerative disorder of early childhood-onset with severe axonal polyneuropathy

**DOI:** 10.1186/s40478-021-01277-5

**Published:** 2021-12-18

**Authors:** Aristides Hadjinicolaou, Kathie J. Ngo, Daniel Y. Conway, John P. Provias, Steven K. Baker, Lauren I. Brady, Craig L. Bennett, Albert R. La Spada, Brent L. Fogel, Grace Yoon

**Affiliations:** 1 Division of Neurology, Department of Paediatrics, The Hospital for Sick Children, University of Toronto, Toronto, ON Canada; 2 Department of Neurology, David Geffen School of Medicine, University of California Los Angeles, Los Angeles, 90095 USA; 3 Department of Pathology and Molecular Medicine, Faculty of Health Sciences, McMaster University, Hamilton, ON Canada; 4 Department of Medicine, Divisions of Physical Medicine and Neurology, McMaster University, Hamilton, ON Canada; 5 Department of Pediatrics, McMaster University Medical Centre, Hamilton, ON Canada; 6 Department of Pathology and Laboratory Medicine, University of California, Irvine, USA; 7 Department of Neurology and Department of Biological Chemistry, UC Institute for Neurotherapeutics, University of California, Irvine, USA; 8 Departments of Neurology and Human Genetics, David Geffen School of Medicine, Clinical Neurogenomics Research Center, University of California Los Angeles, 695 Charles E. Young Dr. South, Gonda Room 6554A, Los Angeles, CA 90095 USA; 9 Division of Clinical and Metabolic Genetics, Department of Paediatrics, The Hospital for Sick Children, University of Toronto, 555 University Avenue, Toronto, ON M5G 1X8 Canada

**Keywords:** SETX, Senataxin, Axonal neuropathy, Neurodegeneration

## Abstract

Pathogenic variants in *SETX* cause two distinct neurological diseases, a loss-of-function recessive disorder, ataxia with oculomotor apraxia type 2 (AOA2), and a dominant gain-of-function motor neuron disorder, amyotrophic lateral sclerosis type 4 (ALS4). We identified two unrelated patients with the same de novo c.23C > T (p.Thr8Met) variant in *SETX* presenting with an early-onset, severe polyneuropathy. As rare private gene variation is often difficult to link to genetic neurological disease by DNA sequence alone, we used transcriptional network analysis to functionally validate these patients with severe de novo SETX-related neurodegenerative disorder. Weighted gene co-expression network analysis (WGCNA) was used to identify disease-associated modules from two different ALS4 mouse models and compared to confirmed ALS4 patient data to derive an ALS4-specific transcriptional signature. WGCNA of whole blood RNA-sequencing data from a patient with the p.Thr8Met *SETX* variant was compared to ALS4 and control patients to determine if this signature could be used to identify affected patients. WGCNA identified overlapping disease-associated modules in ALS4 mouse model data and ALS4 patient data. Mouse ALS4 disease-associated modules were not associated with AOA2 disease modules, confirming distinct disease-specific signatures. The expression profile of a patient carrying the c.23C > T (p.Thr8Met) variant was significantly associated with the human and mouse ALS4 signature, confirming the relationship between this *SETX* variant and disease. The similar clinical presentations of the two unrelated patients with the same de novo p.Thr8Met variant and the functional data provide strong evidence that the p.Thr8Met variant is pathogenic. The distinct phenotype expands the clinical spectrum of *SETX*-related disorders.

## Introduction

*SETX* encodes for senataxin, an RNA/DNA helicase with multiple critical roles including transcriptional regulation, RNA processing, maintenance of genome integrity and the DNA damage response, neurogenesis, regulation of autophagy, and antiviral response [11, 20, 35, 39]. Two well-described neurodegenerative phenotypes have been associated with pathogenic variants in *SETX*: autosomal recessive ataxia with oculomotor apraxia type 2 (AOA2; also known as Spinocerebellar Ataxia with Axonal Neuropathy Type 2, SCAN2), and an autosomal dominant juvenile-onset form of motor neuron disease, Amyotrophic Lateral Sclerosis Type 4 (ALS4) [5, 15].

Amyotrophic lateral sclerosis (ALS) is the prototypical motor neuron disease affecting both upper and lower motor neurons, leading to progressive muscular weakness and atrophy. ALS4 is a familial form of motor neuron disease originally reported in a large Maryland kindred in 1998, and was eventually found to be caused by a heterozygous missense p.Leu389Ser pathogenic variant in *SETX* [9, 10]. The exact mechanism of motor neuron toxicity remains unknown [4]. To date, seven unrelated ALS4 families with four missense pathogenic variants (p.Leu389Ser, p.Thr3Ile, p.Arg2136His, and p.Met386Thr) in the *SETX* gene have been described [2, 9, 10, 33, 42, 46].

In contrast to classic ALS, ALS4 is milder and characterized by earlier onset of symmetrical distal muscle weakness and atrophy, pyramidal signs in the absence of sensory deficits, and an autosomal dominant pattern of inheritance. All previously described patients developed symptoms during the second decade (range 6–22, mean 12.5 years) except for two asymptomatic females. The first symptom was often lower limb weakness, especially of the tibialis anterior [21, 30]. Other frequent symptoms included distal upper extremity weakness and atrophy, hyperactive deep tendon reflexes, clonus, Babinski or Hoffmann signs. Of note, none of the previously described patients had abnormal cognition, cranial nerve abnormalities, sensory symptoms or foot deformities [38].

We describe two unrelated patients with the same de novo c.23C > T (p.Thr8Met) variant in *SETX.* Both patients presented with an earlier onset and more severe clinical phenotype than those previously reported with ALS4. This rare variant was not found in the Genome Aggregation Database (gnomAD) [24] or the large ExAC database of healthy individuals [31] and while predicted to have a deleterious effect by in silico prediction programs, the clinical relevance was difficult to evaluate.

Previously, weighted gene co-expression network analysis (WGCNA) was used to demonstrate that phenotypic differences between AOA2 and ALS4 were associated with distinct gene expression profiles due to mutation-specific changes in the function of *SETX* [15]. WGCNA was also able to identify discrete gene modules defining an AOA2 disease-specific transcriptional signature that could be used to specifically distinguish patients from unaffected carriers [15]. In this study, we used transcriptional profiling to identify an ALS4 disease-specific transcriptional signature using two different ALS4 mouse models and members of a verified ALS4 family. This signature was found to match that of the patients whom we describe with the de novo c.23C > T (p.Thr8Met) variant. These results demonstrate that this SETX-specific transcriptional signature can be used to functionally identify and diagnose patients.

## Case presentation

### Patient 1

Patient 1 was born following a term spontaneous vaginal delivery. Family history was non-contributory, other than two older siblings with mild autistic features. He came to neurological attention at two years of age due to motor delays, arthrogryposis, scoliosis, recurrent pneumonias, and dysarthria.

Developmentally, he was able to turn pages of a book using gross hand movement, but had no pincer grasp, and was unable to write, colour or point. Speech was notable only for severe dysarthria. Regarding activities of daily living, he was able to use a fork with assistance, was toilet-trained, but fully dependent for dressing. At last clinical assessment at age 10 years, he was in a regular grade 5 classroom with an individualized education plan for physical accessibility. He received physiotherapy, occupational therapy, speech and language therapy and daily cough assist therapy. He ambulated using a walker until the age of nine years, when he became fully wheelchair-dependent.

**Fig. 1:**
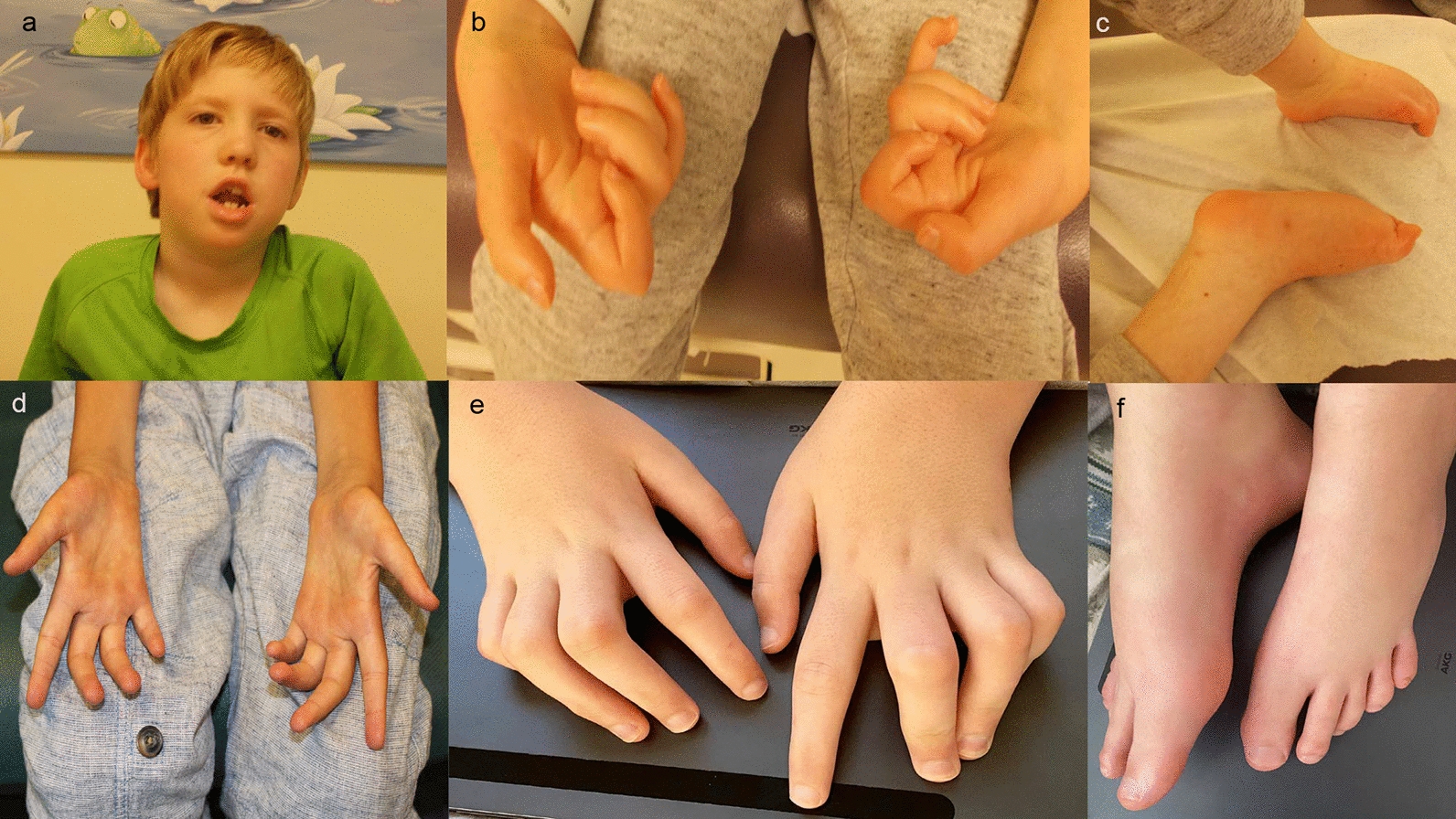
Clinical Photographs of both patients with de novo p.Thr8Met *SETX* variants. Patient 1 at age 9 years (**a**–**c**) and Patient 2 at age 15 years (**d**–**f**)

On physical examination at age 10 years, he was normocephalic with height and weight below the 1st percentile. He had an elongated face, prominent overcrowded dentition and tongue atrophy. He had tapering fingers with contractures of all joints of the hands bilaterally, as well as scoliosis. (Fig. 1a–c) Cranial nerve examination revealed lower facial weakness with sialorrhea and tongue fasciculations. Motor examination revealed reduced muscle bulk throughout, with atrophy of thenar and hypothenar eminences. There was significant axial hypotonia with inability to sit unsupported for longer than 20 s, and appendicular hypertonia of the lower extremities with significant ankle stiffness requiring ankle–foot orthoses. He had generalized weakness, which was most severe distally, and was non-ambulatory. Sensation was difficult to assess, however vibration sense appeared absent. Deep tendon reflexes were absent with bilateral extensor plantar responses.

**Fig. 2:**
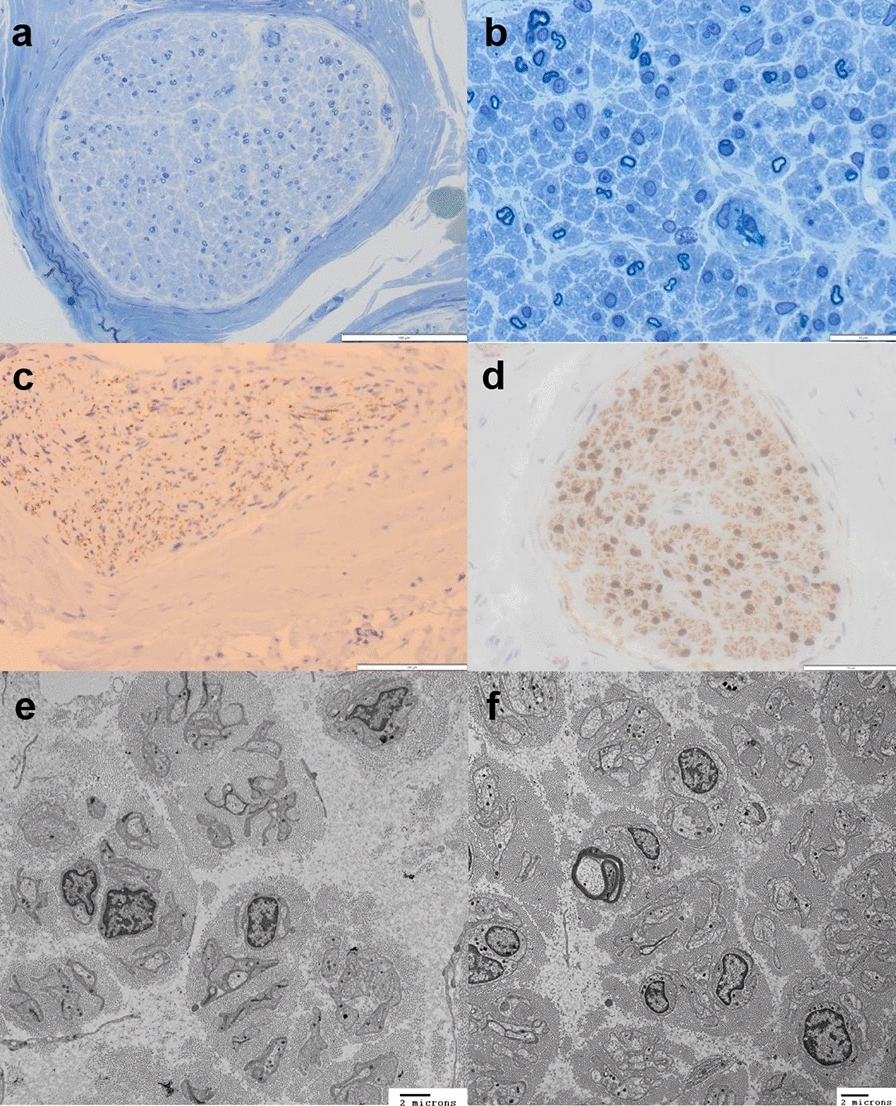
Sural nerve biopsy from Patient 1. Toluidine blue stained sections of the nerve (**a**,**b**) in transverse orientation highlight the moderate to marked loss of larger myelinated axons. There was an absence of inflammation and no onion bulbs. The epineurium/perineurium were unremarkable. Neurofilament immunohistochemistry highlights the loss of larger axons and the relative preservation of the smaller non-myelinated fibres. (**c**) S100 IHC stain revealed mildly reduced Schwann cell nuclei. (**d**) Ultrastructural examination by transmission electron microscopy (**e**,**f**) highlights the paucity of myelinated axons, endoneurial collagenosis, numerous bands of Bugner and atrophy of one residual myelinated fibre (**f**)

Nerve conduction studies at two years of age revealed a severe neuropathy with slowed conduction velocities. Repeat nerve conduction studies at nine years of age revealed absent sensory responses and severely reduced amplitudes of the compound muscle action potentials (CMAPs) with markedly slowed conduction velocities (Table 1). Sural nerve biopsy performed at age four years demonstrated significant abnormalities of the endoneurial compartment in all fascicles. The main finding was a loss of many (> 80%) of the larger myelinated axons (Fig. 2a, b, e, f). Small myelinated fibres and non myelinated axons were better preserved (Fig. 2c). There was absence of myelin remodeling, myelin breakdown material and Schwann cell hyperplasia/onion bulb formation. This in conjunction with the ultrastuctural findings suggested a primary degenerative process ie. axonopathy/neuronopathy. Consistent with the latter is the apparent mild loss of Schwann cells (Fig. 2d). The epineurium and perineurium were normal and there was no inflammation. There was no abnormal storage material detected, although the endoneurial collagen was increased consistent with secondary fibrosis (Fig. 2e). The absence of large myelinated axons, and not de/remyelination, is the likely cause of the observed slowed conduction velocities, as previously reported for *NEFL* mutations [22, 48].

**Table 1:** Nerve conduction studies for both patients with de novo p.Thr8Met *SETX* variants

	Distal latency (normal ≤ ms)	Amplitude (normal ≥ mV)	Conduction velocity (normal ≥ m/s)
*Patient 1 (age 2y)*			
R median motor	9.2 ms (4.3)	0.2 mV (4.0)	14.9 m/s (40)
L median motor	9.2 ms (4.3)	0.5 mV (4.0)	8.5 m/s (40)
*Patient 1 (age 9y)*			
R median motor	9.3 ms (4.4)	0.2 mV (4.0)	8.1 m/s (50)
L median motor	9.2 ms (4.4)	0.1 mV (4.0)	11.7 m/s (50)
R median sensory	NR (3.1)	NR (20)	NR (50)
*Patient 2 (age 7y)*			
L median motor	4.5 ms (4.4)	0.5 mV (4.0)	25 m/s (50)
L tibial motor	NR (5.0)	NR (4.0)	NR (40)
L peroneal motor	NR (6.5)	NR (2.0)	NR (40)
L median sensory	NR (3.1)	NR (20)	NR (50)
L superficial peroneal sensory	NR (4.4)	NR (6)	NR (40)
*Patient 2 (age 8y)*			
L median motor	5.1 ms (4.4)	0.8 mV (4.0)	29.5 m/s (50)
R median motor	6.2 ms (4.4)	0.8 mV (4.0)	19.0 m/s (50)
L ulnar motor	5.1 ms (3.3)	0.3 mV (6.0)	27.1 m/s (50)
L tibial motor	NR (5.0)	NR (4.0)	NR (40)

*NR* Non-Recordable, *L* Left, *R* Right

Trio whole exome sequencing of the proband and his parents (GeneDx) revealed a heterozygous de novo variant of uncertain significance (VUS) in *SETX*, c.23C > T (p.Thr8Met). Whole genome sequencing, coupled with RNA sequencing in blood (Medical Neurogenetics), revealed the same de novo variant in *SETX*. No other clinically relevant variants, including in *NEFL*, were identified on either study.

### Patient 2

Patient 2 was born at term following a normal pregnancy, via emergency C-section due to fetal distress, with a subsequent unremarkable perinatal course. There was no significant family history.

He presented with significant infantile-onset hypotonia and delays in gross motor milestones. He achieved independent ambulation after 14 months of age, however experienced rapid progression of his neurological symptoms between 8 and 10 years of age, with worsening muscle weakness and atrophy, and severe contractures of the fingers, becoming wheelchair-dependent by age 12 years. He had increasing feeding difficulties and eventually underwent G-tube insertion at age 12. He required nocturnal C-PAP therapy due to decreased respiratory function. Academically, at last assessment at age 14 years, he was performing well in grade 9. He was fully dependent for all activities of daily living, required 24-h care, and received physiotherapy, occupational therapy and had a personal support worker.

On physical examination at age 14 years, he had marked thoracic scoliosis, scapular winging and significant finger, knee and ankle contractures (Fig. 1d–f). On neurological examination, cranial nerve assessment demonstrated myopathic facies, dysarthric speech, as well as tongue fasciculations and atrophy. Motor examination revealed mild appendicular hypertonia, as well as profound generalized muscle atrophy, and significant lower extremity weakness, with inability to bear weight and raise his arms above his shoulders. Deep tendon reflexes were 1 + bilaterally. Plantar responses were extensor and while sensation to pain and light touch were preserved, he had decreased vibration and proprioception sensation.

Nerve conduction studies at age 7 years and repeated at 8 years revealed absent sensory responses and severely reduced amplitudes of the CMAPs with slowed conduction velocities (Table 1).

Trio whole exome sequencing (GeneDx) revealed a heterozygous de novo p.Thr8Met variant (c.23 C > T) in *SETX*.

Multiple bioinformatics models predict a high likelihood of damaging effect for the (c.23 C > T (p.Thr8Met) variant. The PolyPhen score is 0.998 on a scale of 0–1, where 1 is highly deleterious. The SIFT score is 0.0 on a scale of 0–1, where zero is highly deleterious and 1 is benign [34]. This variant was also predicted to be pathogenic by MutationTaster2 [44].

Additional details of both patients are summarized in Table 2.

**Table 2:** Detailed clinical characteristics of patients with de novo p.Thr8Met *SETX* variants. NR: not reported

	Current study	Current study	Individual described by Kitao et al., 2020
Patient	Patient 1	Patient 2	Proband
Age at last review	10 years	14 years	29 years
Gender	Male	Male	Male
Country of parental origin	Mother: Irish Father: Irish-Canadian	Mixed Northern European-Italian	Japan
Birth weight/gestational age	3.2 kg / 41 weeks	3.3 kg / 40 weeks	NR
Current height (centile)	< 1%ile	133 cm (< 1%ile)	130 cm (< 1%ile)
Current weight (centile)	19.5 kg (< 1%ile)	30 kg (< 1%ile)	21 kg (< 1%ile)
Feeding/swallowing difficulties	Failure to thrive from an early age	Failure to thrive and diarrhea (age 4)	NR
Respiratory function	Recurrent episodes of croup, recurrent pneumonias, moderate obstructive sleep apnea	Nocturnal CPAP, restrictive lung disease	Respiratory failure at age 20 requiring tracheostomy with positive-pressure ventilation (TPPV)
G-tube and age at dependency	No	Age 12	NR
Developmental/academic history	Grade 5, above average	Grade 9, grade A average	NR
Communication	Excellent vocabulary and spelling, severe dysarthria	Excellent written communication, severe dysarthria	No dysarthria
Gross motor	Sat 7–8 months, crawled 18 months, cruised 2 years, ambulated with walker until 9 years	Delays in rolling, sitting, crawling, standing, walked at 14 months, ambulated until 12 years	Walked at 2.5 years, ambulated until 12 years
Fine motor	No pincer grasp, severely limited by hand contractures	Severely limited by hand contractures	Difficult to assess due to muscle weakness and contractures
Functional status	Non-ambulatory (wheel-chair dependent), can use fork with assistance, fully dependent for most ADLs	Non-ambulatory (wheel-chair dependent), fully dependent for ADLs, required 24-h care	Non-ambulatory at 12 years
Facial weakness	Dysarthria, lower facial weakness	Myopathic facies, mild bilateral ptosis, dysarthric speech	Weakness of facial muscles
Tongue fasciculations	Yes	Yes (with associated atrophy)	No
Axial hypotonia	Significant (unable to sit unsupported for 20 s)	Significant (unstable when leaning forward in his chair)	Decreased tone
Appendicular hypertonia/spasticity	Moderate in legs bilaterally	Mild	NR
Muscle weakness	Generalized weakness, distal more than proximal	Generalized weakness, distal more than proximal	Distal muscle weakness
Muscle atrophy	Generalized atrophy, most notable of thenar and hypothenar eminences	Generalized atrophy, most notable in fingers	Generalized muscle atrophy
Sensory exam	Intact vibration, proprioception mildly decreased	Pain and temperature preserved, vibration and proprioception mildly decreased	Impairment in all modalities distally
Deep tendon reflexes	Absent throughout	1 + throughout	Absent throughout
Extensor plantar response	Bilaterally extensor	Bilaterally extensor	Absent
Scoliosis	Severe scoliosis with a Cobb angle of 75 degrees	Marked left-sided thoracic scoliosis	Severe scoliosis, Cobb angle of 71 degrees
Contractures	Bilateral club feet at birth, severe hand contractures	Severe finger, knee, ankle contractures	Hand contractures
Cardiac function	Echocardiogram (age 9): normal	Normal	NR
Other	CSF, CPK, serum neuromuscular antibody panel, *PMP22*, *MPZ, ERG2, LITAF, PRX, NFL* sequencing, chromosomal microarray, and mitochondrial DNA sequencing: negative	Karyotype, Fragile X, FISH and methylation studies for Prader-Willi and Angelman syndromes, CPK, sequencing of *PRX, PMP22, EGR2, MPZ* and *GJB1*: negative	Comprehensive gene analysis of hereditary peripheral neurological diseases by next-generation sequencing: negative
Brain MRI	Several non-specific tiny white matter hyperintensities in the centrum semiovale and peritrigonal regions bilaterally	Non-specific left peritrigonal increased T2 and FLAIR signal	Normal
Spine MRI	Normal	Normal	NR
EMG/NCS	Severe axonal loss and slowed conduction velocity (Table 1)	Severe axonal loss and slowed conduction velocity (Table 1)	Decreased compound muscle action potential amplitudes and reduced motor nerve conduction velocity with absent sensory nerve action potentials
*SETX* (NM_015046.5)	c.23C > T (de novo)	c.23C > T (de novo)	c.23C > T (de novo)
Genomic position (Hg19)	chr9:135224793G > A	chr9:135224793G > A	chr9:135224793G > A
Predicted effect on protein	p.Thr8Met	p.Thr8Met	p.Thr8Met
Type of mutation	Missense	Missense	Missense

### ALS4 mouse models

Transgenic wild type mice, ALS4 transgenic mice with p.Arg2136His mutation, and ALS4 knock-in mice with p.Leu389Ser mutation were generated as previously described [4]. Briefly, full-length human *SETX* (untagged) was expressed in WT-SETX and R2136H-SETX transgenic mice from the mouse prion promoter (mo.PrP). Strong expression within the neuromuscular axis results from use of this promoter. Given that SETX protein is maintained at tightly controlled levels [6, 13], total protein levels were not elevated for combined mouse Setx and human SETX. Specifically, Arg2136His mutant SETX was determined to represent ~ 71% of total SETX protein in transgenic line #1920 that was used in this study. For the *Setx* knock-in model, a standard gene-targeting approach was employed to introduce the well characterized Leu389Ser mutation. In SETX ^L389S+/−^ mice, SETX protein levels were comparable with non-transgenic littermates, validating that the Leu389Ser isoform was expressed at physiologic levels. By careful characterization with a range of neuromuscular tests, we concluded that these complementary transgenic and gene-targeted models effectively recapitulate human ALS4 disease [4]. ALS4 SETX mice develop neuromuscular phenotypes and motor neuron degeneration with key ALS features of TDP-43 nuclear clearance and cytosolic mislocalization. These findings are consistent with ALS4 patient pathology, and the observed normal life-span of these mice was also consistent with ALS4 human patients.

All experiments were performed following the National Institutes of Health animal protection guidelines, and approved by the Animal Research Committee.

### Microarray and data processing

Total RNA extracted from mouse cerebellar tissue were analyzed using MouseRef-8 v2.0 expression arrays per the manufacturer’s instructions. Data was normalized and filtered for batch effects using the limma software package [41].

### RNA-sequencing, data processing, and differential gene expression analysis

RNA-sequencing was carried out using the Illumina TruSeq Stranded RNA library kit with RiboZero Gold treatment with 75 bp paired-end reads. Sequencing reads were trimmed for adapters with Trimmomatic and subsequently mapped with the STAR spliced read aligner to the GRCh38 human reference genome and Gencode GTF (v27) [8, 14, 16]. HTSeq was used to generate read counts. Log RPKM values were generated by using custom script in R based on the GenomicFeatures library and rtracklayer library [28, 29]. Differential gene expression analysis was performed on unaffected parental controls and affected patients using DESeq2 [1]. The *SETX* control patient was not included in this analysis to reduce the introduction of background. FDR < 0.05 and |log2FC|> 0.3 thresholds were applied.

### Weighted gene co-expression network analysis (WGCNA)

In contrast to the assessment of individual gene expression levels, Weighted Gene Co-expression Network Analysis (WGCNA) is designed to identify sets of genes whose expression is co-regulated, and thus represent gene networks of shared or overlapping biological functions. In this study, WGCNA was conducted as previously described [26]. Briefly, gene expression datasets were correlated to construct gene co-expression networks. Modules, groups of genes that were co-expressed and clustered together, were identified. Key modules that were found to be correlated with ALS4 disease phenotypes were interrogated. Gene ontology analysis was performed using DAVID Bioinformatics resources (NIAID, NIH) on modules that had gene expression profiles correlated with disease [23]. Statistical comparisons between module gene membership were calculated using hypergeometric probability. Cell type enrichment studies in mice data were determined from published datasets [45, 49]. Module preservation between datasets was calculated by WGCNA’s module preservation function [27]. Samples that appeared to be outliers were excluded from analysis. WGCNA’s built in function empiricalBayesLM was used to remove covariates that were present in patient data.

WGCNA was conducted on microarray expression data from RNA obtained from spinal cord and cerebellum of two different ALS4 mouse models (representing p.Arg2136His and p.Leu389Ser respectively) and wild type transgenic control mice (three replicates of each condition) to identify modules of co-expressed genes correlated with the ALS4 disease phenotype and thus represents the disease-specific transcriptional signature [4]. Cerebellar WGCNA identified two modules that correlated with disease status, ALS4MmMod02 (543 genes) and ALS4MmMod32 (185 genes). There was no significant overlap between these two modules compared to previously published AOA2 disease-specific modules (mouse cerebellum: green/blue, human peripheral blood: blue and turquoise) [15]. Both ALS4MmMod02 and ALS4MmMod32 modules were not preserved in the AOA2 disease-specific modules indicating both modules are ALS4 disease-specific (data not shown). Cell type enrichment analysis showed that ALS4MmMod02 was enriched for neuronal markers (*p* = 8.81 × 10^–19^) and ALS4MmMod32 was mildly enriched for microglia markers (*p* = 0.03). GO analysis of the ALS4MmMod02 module showed enrichment of genes involved in synaptic signaling, including the highly correlated “hub” gene *CCKBR*, nervous system development, regulation of DNA/RNA metabolic process, and gene expression (Table 3). Genes from the ALS4MmMod32 module were involved in diencephalon development, forebrain morphogenesis, muscle organ development, nervous system development, and regulation of gene expression which included the “hub” gene *ANK2* (Table 3).

**Table 3:** Top 5 most significant and *SETX* function-related Gene Ontology (GO) categories for ALS4 disease-associated WGCNA modules

Module name	Gene ontology category
ALS4MmMod02 overall	1. GO:0099536 ~ synaptic signaling
	2. GO:0007399 ~ nervous system development
	3. GO:0031223 ~ auditory behavior
	4. GO:0046903 ~ secretion
	5. GO:0032990 ~ cell part morphogenesis
ALS4MmMod02 *SETX* function	1. GO:0007218 ~ neuropeptide signaling pathway
	2. GO:0006915 ~ apoptotic process
	3. GO:0051254 ~ positive regulation of RNA metabolic process
	4. GO:0010628 ~ positive regulation of gene expression
	5. GO:0006259 ~ DNA metabolic process
ALS4MmMod32 overall	1. GO:0021536 ~ diencephalon development
	2. GO:0051271 ~ negative regulation of cellular component movement
	3. GO:0007159 ~ leukocyte cell–cell adhesion
	4. GO:0022409 ~ positive regulation of cell–cell adhesion
	5 GO:0010604 ~ positive regulation of macromolecule metabolic process
ALS4MmMod32 *SETX* function	1. GO:0048853 ~ forebrain morphogenesis
	2. GO:0007517 ~ muscle organ development
	3. GO:0007399 ~ nervous system development
	4. GO:0010468 ~ regulation of gene expression
	5. GO:0051254 ~ positive regulation of RNA metabolic process
ALS4HsMod77 overall	1. GO:0015031 ~ protein transport
	2. GO:0043067 ~ regulation of programmed cell death
	3. GO:0034308 ~ primary alcohol metabolic process
	4. GO:0008299 ~ isoprenoid biosynthetic process
	5. GO:0051046 ~ regulation of secretion
ALS4HsMod77 *SETX* function	1. GO:0050804 ~ modulation of synaptic transmission
	2. GO:0006915 ~ apoptotic process
	3. GO:0006887 ~ exocytosis
	4. GO:0045773 ~ positive regulation of axon extension
	5. GO:0050684 ~ regulation of mRNA processing

**Fig. 3:**
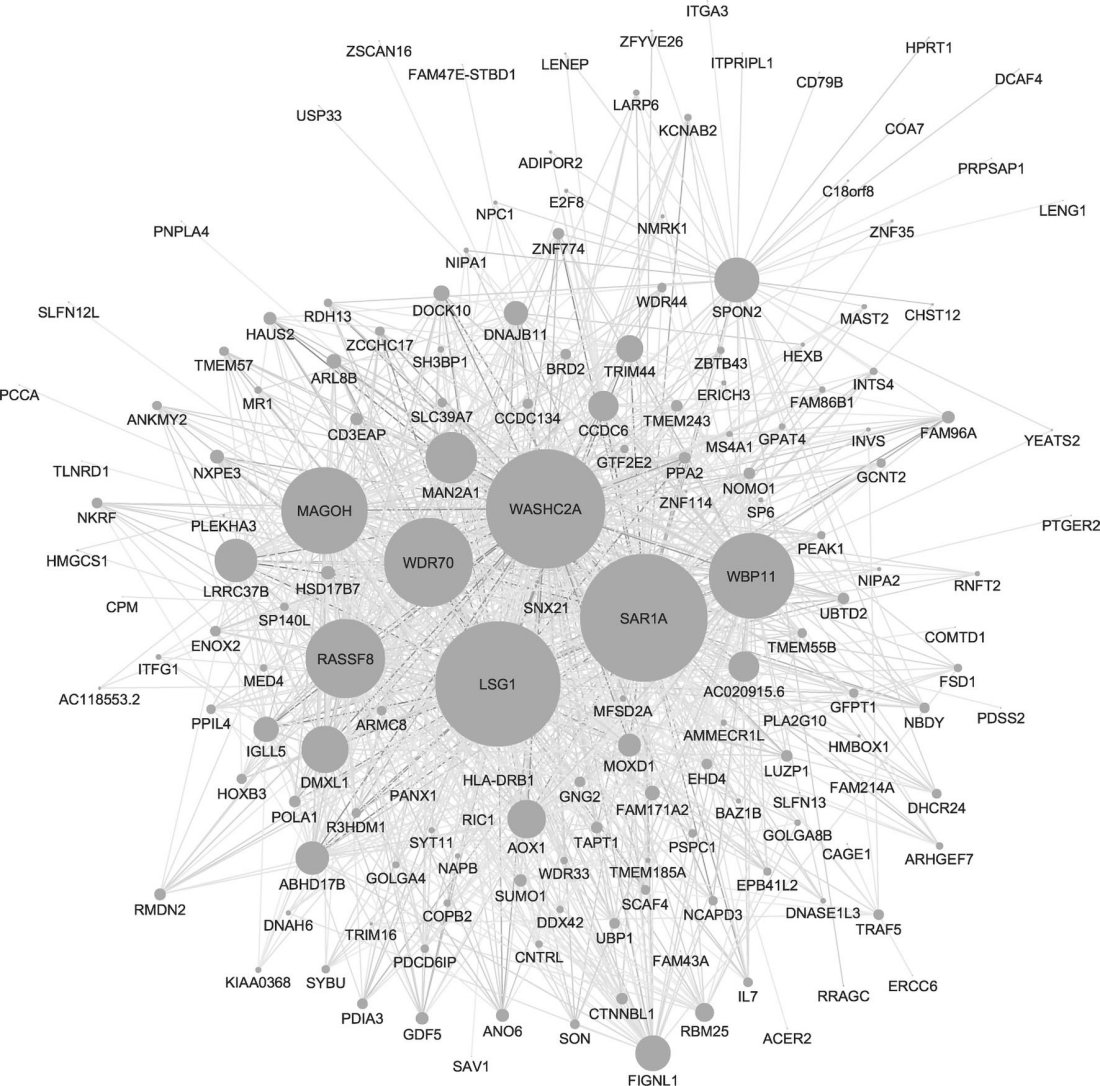
ALS4 patient module ALS4HsMod77. Top 1000 connections are shown. Node size reflects degree of network connectivity

To determine if this murine ALS4 disease-associated expression signature could be used to diagnose ALS4 patients, WGCNA was used to compare whole blood expression data from two related patients with confirmed ALS4 (p.Leu389Ser mutation) [4, 10]. As expected, differential gene expression analysis of ALS4 and control patients indicated that *SETX* was not differentially expressed. To identify modules that were ALS4 disease-associated, we evaluated the module eigengene relationship between ALS4 patients and controls, and identified seven modules which strongly correlated with disease status. None of these modules showed any significant gene membership with module ALS4MmMod02, however one module, ALS4HsMod77 (467 genes), had a significant relationship with module ALS4MmMod32 (*p* = 0.04) indicating that ALS4 *SETX* mutations in both mice and humans lead to disruption of this same network. Shared genes between ALS4MmMod32 and ALS4HsMod77 include *HEXB* and *ALOX12,* both of which are involved in regulation of gene expression and nervous system development, with the latter also involved in programmed cell death and apoptosis. GO analysis of genes in ALS4HsMod77 also showed association with protein transport including three hub genes (*LSG1, SAR1A,* and *WBP11*), regulation of programmed cell death, apoptotic processes, modulation of synaptic transmission, regulation of axon extension, and regulation of mRNA processing (Table 3, Fig. 3). Comparison with ALS4MmMod32 suggests these modules may represent biological pathways related to senataxin function (Table 3). In contrast to the mouse modules, while there was no significant overlap between the ALS4HsMod77 module compared to the previously published mouse AOA2 disease-specific module (mouse cerebellum: green/blue), there was a significant overlap with one of the two human AOA2 disease-specific modules (peripheral blood: turquoise; *p* = 6.04 × 10^–05^) [15].

WGCNA data from the whole blood of Patient 2 and a control patient who had a distal motor neuropathy and a distinct rare variant in *SETX* (p.Gly2172Asp), were compared to the ALS4 disease-specific signature derived from mouse and human as defined above*.* Bioinformatic predictor analysis indicated that p.Gly2172Asp was consistent with a polymorphism, whereas p.Thr8Met was consistent with a disease-causing pathogenic variant (Ensembl Variant Effect Predictor, MutationTaster2) [44]. To minimize covariates from family specific background, expression data from patients was first normalized to baseline expression in a respective control family member (one control per family). We then compared the modules from each family to module ALS4HsMod77 to evaluate variant pathogenicity (Table 4). The positive controls (p.Leu389Ser ALS4 verified patients) showed significant association with module ALS4HsMod77 (*p* = 0.02). The p.Thr8Met patient (Patient 2) also showed significant association with this module (*p* = 0.01), supporting pathogenicity of this variant. In contrast, the p.Gly2172Asp patient did not show a significant association with the ALS4HsMod77 module (*p* = 0.99), consistent with the bioinformatic prediction of this variant being a benign polymorphism. Given this new functional evidence, we consider the p.Thr8Met *SETX* variant to be pathogenic based on ACMG criteria [40].

**Table 4:** Patient variant associations to ALS4 disease-associated modules

Comparisons	ALS4HsMod 77
ALS4 Patients (n = 2) versus Controls (n = 3)	**0.02**
Patient 2 (n = 1) versus Controls (n = 3)	**0.01**
Control *SETX* (n = 1) versus Controls (n = 3)	0.99

Significant *p*-values in bold. Patient 2 carries the p.Thr8Met variant reported here while Control *SETX* carries a p.Gly2172Asp variant, bioinformatically predicted to be benign

## Discussion and conclusions

Clinically, the phenotype of ALS4 overlaps with hereditary spastic paraplegias (HSP), distal hereditary motor neuropathies (dHMN), and hereditary peripheral polyneuropathies [9, 10, 12]. All of these entities are characterized primarily by predominantly distal muscle weakness and atrophy, accompanied by pyramidal signs, with variable age at onset of symptoms (but generally before 25 years) [9, 10, 12]. In general, they are characterized by a slow rate of progression with a normal life span but significant functional impairment, usually occurring in the fifth or sixth decades of life [9, 10].

We describe the clinical features of two unrelated patients with neuromuscular symptoms considerably more severe than those typically associated with ALS4. In both our patients with de novo* SETX* c.23C > T (p.Thr8Met) variants, we note several marked differences from the previously described ALS4 phenotype. Both patients showed an earlier age of onset, developing symptoms in the first decade as opposed to the second decade, as is more commonly seen in ALS4. Significant failure to thrive requiring medical intervention and support was noted, also distinct from the typical ALS4 presentation. Other notable features included early musculoskeletal involvement with severe contractures and scoliosis as well as cranial nerve and bulbar dysfunction, demonstrated by evidence of myopathic facies, tongue fasciculations, significant sialorrhea and episodes of aspiration. Electrophysiologically, a severe sensorimotor polyneuropathy was observed, which has not been previously described in ALS4 patients. The severe reduction in nerve conduction velocities observed in our patients was suggestive of a hypomyelinating neuropathy such as Dejerine-Sottas Syndrome. Most often, motor conduction studies in patients with confirmed ALS4 were either normal or revealed mild reduction in compound muscle action potential (CMAP) amplitudes [10, 30]. Lei et al. (2020) showed that conduction blocks (CBs) and abnormal temporal dispersion (TD) were also possible in patients with ALS4. This was thought to be possibly associated with the expression of senataxin in peripheral nerves [30]. To our knowledge, there has been no prior report of any sensory nerve conduction abnormalities reported in ALS4 patients. The nerve biopsy of patient 1 confirmed axonal degeneration rather than a primary demyelinating neuropathy as suggested by the nerve conduction studies, and the markedly slowed nerve conduction velocities observed are likely due to the absence of large myelinated axons. Finally, both patients showed rapid progression, leading to significantly impaired ambulation and wheelchair-dependence by age 12 years or earlier.

Given the rarity of the disease and the limited number of patients, it is challenging to definitively prove this variant may confer a more severe phenotype than previously described for ALS4. However, in the literature there is another clinical report, which was published during finalization of the current study, of a patient with this same de novo variant and a similar clinical presentation [25]. This patient became symptomatic at 2 years of age and became non-ambulatory at 12 years. He experienced respiratory failure at the age of 20 requiring tracheostomy with positive-pressure ventilation (TPPV). On clinical examination, he showed distal muscle weakness and generalized muscle atrophy, including the facial muscles, scoliosis, and a peripheral sensory neuropathy (Table 2). Nerve conduction studies showed a decrease of compound muscle action potential amplitudes and a reduction of motor nerve conduction velocity with absent sensory nerve action potentials. As this current study now functionally validates p.Thr8Met as the causative *SETX* pathogenic variant in this patient, and given that the ancestry of the patient is Japanese, this further extends the worldwide prevalence of this disorder in addition to expanding the phenotypic spectrum.

The complexity of clinical features observed in patients with pathogenic variants arising de novo in genes typically associated with dominantly inherited neurological disorders, such as channelopathies and hereditary spastic paraplegias, is becoming increasingly recognized [7, 17, 18, 32, 47]. Several studies have detailed the earlier age at symptom onset and greater severity of clinical symptoms in patients with de novo variants in *SPAST* [37, 43], compared to patients from SPG4 families with inherited *SPAST* variants. It appears that the de novo p.Arg499His *SPAST* pathogenic variant may represent a recurrent cause of severe early-onset SPAST-related neurodegenerative disorder which is distinct from typical SPG4 [19, 36]. The p.Thr8Met variant in *SETX* may reflect a similar phenomenon; however, larger cohorts of patients with pathogenic variants in *SETX* will be required to confirm this.

Private variation limited to single families and often to only one affected family member is difficult to link to neurological diseases based on sequence alone, highlighting a need for functional and other confirmatory studies. Both patients we describe were found to have a de novo p.Thr8Met variant in *SETX*, strongly suggesting this was the cause of their similar phenotypes. To validate pathogenicity of this variant, we used transcriptional profiling from two different ALS4 mouse models and affected members of a genetically-confirmed ALS4 family to identify an ALS4 disease-specific transcriptional signature by WGCNA. We observed that the transcriptional signature of one of our p.Thr8Met patients significantly matches this profile, demonstrating that this ALS4 disease-specific transcriptional signature can be used to functionally identify and diagnose patients with *SETX*-mediated neurodegenerative disease. One limitation of this study is that while we profiled both spinal cord and cerebellum from the ALS4 mouse models, only WGCNA from the cerebellum produced statistically significant disease-associated networks, likely due to the small sample number and reduced cellular homogeneity inherent in the spinal cord tissue dissection procedure (data not shown). Despite this, the fact that our study involves cross-species comparison between mice and humans and non-target cross-tissue comparison of cerebellum to peripheral blood further underscores the significance of the observed gene expression correlation, reflecting conservation of the genetic signature broadly across both species and tissue. This may reflect a critical biological relevance of the ubiquitously-expressed senataxin, as a similar observation was previously seen across species and tissue for AOA2 as well [3, 15].

Another limitation of this study is the lack of patient data for WGCNA analysis due to the rarity of *SETX*-related neuromuscular disorders and the difficulty in confirming pathogenic variants in affected individuals. However, despite this limitation in statistical power, the observation that module ALS4HsMod77 showed a significant gene membership with module ALS4MmMod32 (*p* = 0.04) across species and tissues is striking and likely reflects a strongly shared underlying molecular pathology, as has been previously described for AOA2 [3, 15]. This reduction in power may have contributed to the observation that none of the other network modules identified in the verified ALS4 family had a significant gene membership with either module ALS4MmMod02 or ALS4MmMod32 derived from the mouse ALS4 models, and that module ALS4HsMod77 did not have any significant gene membership with module ALS4MmMod02. Alternatively, it is possible, in contrast to AOA2, that molecular aspects of the ALS4 disease differ between mice and humans with modules ALS4MmMod02 and the other human modules being unique in their respective species or tissue [3, 15]. The relationship between the ALS4HsMod77 and the AOA2 disease-specific turquoise module is also of interest, as such a relationship was not previously observed in cultured human fibroblasts [14], and may reflect shared disease-related functional relationships of senataxin within patient peripheral blood versus limitations in specificity due to the small sample size in this study. Although the presently defined transcriptional signature provides a framework for distinguishing patients with *SETX*-related neurodegenerative phenotypes, including ALS4 patients, from those with normal senataxin function, further study of additional patients and families will be important to better define the specific molecular pathways disrupted in this spectrum of disease.

## Data Availability

Microarray and sequencing data are available in the National Center for Biotechnology Information (NCBI) Gene Expression Omnibus (GEO) and data processing scripts are available upon request.
